# Optimization of Residual Hexane in Edible Oils Analysis Using Static Headspace Gas Chromatography

**DOI:** 10.1155/2021/1941336

**Published:** 2021-10-29

**Authors:** Azrul Hisyam Samsuri, May Yen Ang, Shean Yeaw Ng

**Affiliations:** ^1^Shimadzu Malaysia Sdn. Bhd, No. 6, Lorong Teknologi 3/4A, Nouvelle Industrial Park 2, Taman Sains Selangor 1, Kota Damansara, 47810 Petaling Jaya, Selangor, Malaysia; ^2^Institute for Tropical Biology and Conservation, Universiti Malaysia Sabah, Jalan UMS, 88400 Kota Kinabalu, Sabah, Malaysia

## Abstract

This study aims to determine the residual hexane in four edible oils in Malaysia using a simple, rapid, and automated method in order to improve the efficiency and productivity of the analysis. Gas chromatography (GC/FID) equipped with a headspace autosampler (HS-20) was used to perform the analysis. Incubation time for each injection was successfully optimized from one hour to 30 minutes (50% reduction) compared to the official AOCS method Ca 3b-87. Out of the four tested edible oils, only the hexane residues detected in sunflower oil exceeded the maximum residue limit (MRL) set by the European Union regulation. Significant difference of the results obtained between large calibration range (0–938 mg kg^−1^) and small calibration range (0–68 mg kg^−1^) suggests that there is a need to use a lower standard calibration concentration to avoid misinterpretation of analysis results. Method validation applies to the technical hexane; 2-methylpentane, 3-methylpentane, cyclohexane, and methylcyclopentane, the signal-to-noise (S/N), as well as the limit of quantification (LoQ) values was found to be 218.20, 221.45, 746.37, 97.37 and 0.85, 0.84, 0.25, 1.93 mg kg^−1^, respectively. Good linearity, repeatability, and low carryover of this method have provided an alternative way to analyze the content of the residual hexane in edible oils in a more efficient manner. Current study might provide a fundamental reference for the improvement of the AOCS official Ca 3b-87 method for determination of hexane residues in fats and oils analysis in the future.

## 1. Introduction

Oil palm, rubber, cocoa, and rice represent the major crops in Malaysia, which have substantial contribution to our economy growth. It is noteworthy that the role of oil palm is particularly important as it accounting 28% of world palm oil production and 33% of world exports. Edible vegetable oils can be derived from the fruits of oil palms. In edible oil production, mechanical pressing and solvent extraction are the two common methods used in the industry, whereby the former method is the most traditional method of oil extraction [1]. About 60–80% of the oil can be obtained by mechanical pressing, and the pressed-cake will be sent to a solvent extraction plant to optimize the oil recovery efficiency ranging from 5–20% [2]. These oils yielded from solvent extraction could be extremely valuable, especially when the crude palm oil prices surge. One of the most effective solvents used for oil extraction is hexane due to its nonpolar nature. However, the commercial hexane used in this production typically consists of some isomers (2-methylpentane, 3-methylpentane, methylcyclopentane, and n-hexane), which are known to have a detrimental health effect [3]. Main toxic effects reported on human studies are muscular weakness, headache, dizziness, and slight nausea [4]. Due to that, according to European Union regulations, maximum residue limit (MRL) of hexane has been set at 1 mg kg^−1^ when it is used in the production or fractionation of fats and oils as well as production of cocoa butter [5]. This regulation has led to various methods being developed for the determination of hexane residue in fats and oils.

Several studies have been carried out on the analysis of residual hexane in food products. Mirghani and Che Man [6] developed a method for the determination of hexane residues in palm and peanut oils based on FTIR spectroscopy with attenuated total reflectance (ATR). Despite of its speed, convenience, and environment friendly, difficulty in interpreting the results have urged most of the industries are still prone to follow the gas chromatography method. The headspace-solid phase microextraction (SPME) method was developed to analyze the hexane residues in olive oil [7]. Similarly, the advantages of short analysis time, without the need of using solvent, are still not able to attract the interest of most industries to adopt this method due to the challenge in terms of collecting the highly concentrated solvent [8]. On the other hand, the AOCS official method Ca 3b-87 derived from International Union of Pure and Applied Chemistry Standard Method (IUPAC) has also evaluated the presence of hexane using HS-GC-FID [9]. This method has been successfully applied and accepted worldwide in the routine analysis of residual solvents in fats and edible oils.

However, preanalysis and postanalysis procedures highlighted in the AOCS official method are manual, labor intensive, time consuming, and hence, susceptible to errors. Several industrial managers have giving out their opinion regarding this matter in a Palm Oil Workshop organized by Shimadzu Sdn Bhd in 2019 (unpublished data). There are increasing numbers of industries looking forward to use an automated system that is not only able to increase productivity but also shorten the analysis time. To the best of our knowledge, there is no study to optimize the official method procedures of hexane residues in edible oil in Malaysia. Hence, current study aims to evaluate the hexane residues of four edible oil samples from the market of Malaysia using a rapid, simple, and automated method.

## 2. Materials and Methods

  General: sunflower oil, corn oil, sesame oil, and palm oil were purchased from hypermarket in Kota Damansara, Malaysia, on March 2019. Headspace crimp vials (20 ml) were purchased from Shimadzu Corporation (Shimadzu Asia Pacific, Singapore). Eppendorf pipettes were purchased from Sigma Aldrich (St. Louis, MO, USA).
  Reagents: HPLC-grade analytical reagents including hexane, hexane isomers, and heptane were obtained from Merck (Germany).
  Instrumentation: GC/FID (GC-2030; Shimadzu, Japan) and the headspace sampler (HS-20; Shimadzu) were used in this study. Helium was used as the carrier gas for the analysis using GC/FID. The column flow was set at 0.8 ml min^−1^ with a flow control mode using constant linear velocity. The detector and injection temperature were set at 120°C with a 1 : 100 split mode.
  Headspace sampler: headspace sampler (HS-20; Shimadzu, Japan) was used in this study. The incubation oven temperature of 80°C, sample line temperature of 90°C, transfer line temperature of 100, agitating level 3, equilibrating time of 30 min, pressurizing time of 1 min, load time of 0.5 min, and injection time of 1 min were configured. For method optimization, incubation time was set ranging from 10 to 60 min at a 10-minute interval.
  Column: SOLGEL-1MS (30.0 m; 0.25 mm ID; 0.25 *μ*m df) was used in this study. The column was maintained at 40°C for 10 min, and then, it was heated up to 100°C at a rate of 30°C min^−1^ and maintained at 100°C for 3 min.
  Standard preparation: standards were prepared using a matrix-match method. 5 g (±0.01 g) of solvent-free edible oils was weighed in a headspace vial. 5 *μ*l of n-heptane (ISTD) was added into the oil. Next, technical hexane was added into the vial, leaving one vial with no added solvent as blank as given in Table 1. Each vial was covered with the headspace cap and tightly crimped. After that, all standards were vortexed for five minutes before being transferred to the headspace sampler for analysis.
  Sample preparation: edible oils available in Malaysia's market were analyzed. 5 g (±0.01 g) of the oils was weighed in the headspace vial. 5 *μ*l of n-heptane (ISTD) was added into oil. The oil was then vortexed for five minutes before being transferred to the headspace sampler for analysis.
  Statistical analyses: all analyses were carried out in triplicates, and the data were statistically analyzed by one-way ANOVA. The level of statistical significance was set at *p* < 0.05 using SPSS software version 23.0.

## 3. Results and Discussion

**Figure 1:**
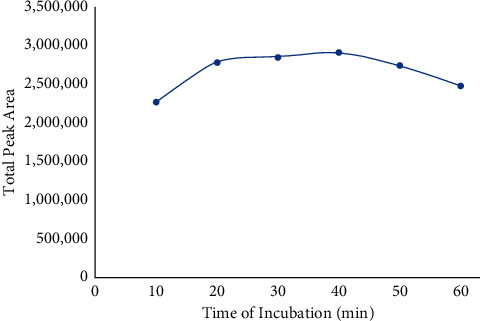
Result of the total peak area of hexane analyzed using different incubation times.

The effect of incubation time was tested to optimize the method. Incubation time ranged from 10 to 60 minutes at a 10-minute interval was studied. Based on the result, 30 minutes of the incubation time was the best for the analysis because there is no significant increase of the total peak area of hexane thereafter as shown in Figure 1. Within this range, headspace extraction has already reached its equilibrium. The result has shortened 50% of the incubation time for each injection as compared to the official AOCS method.

**Figure 2:**
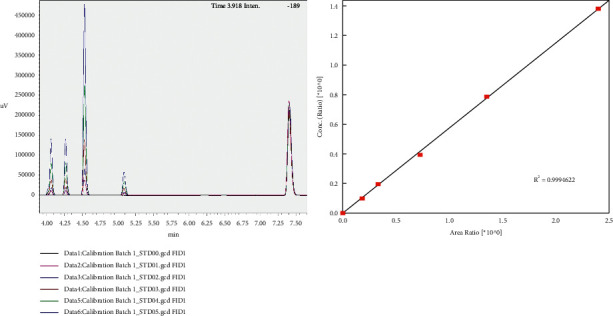
Chromatogram and group-calibration curve of standards (0–938 mg kg^−1^).

Quantification of residual hexane was based on matrix-match calibration and internal standard. The same amount of internal standard was spiked into oil matrix prior to addition of different amount of technical hexane to plot the calibration curve. Chromatogram and the group-calibration curve of the standards are shown in Figure 2. These four peaks are belonging to hydrocarbons that make up the technical hexane. Hydrocarbons that usually make up the technical hexane include 2-methylpentane, 3-methylpentane, cyclohexane, and methylcyclopentane. These four peaks were grouped together to plot a group-calibration curve. The group-calibration curve with good coefficient of determination (*R*^2^) value of more than 0.999 was obtained for these analyzed standards (0–938 mg kg^−1^). Using this calibration curve, residual hexane in four different types of edible oils available in Malaysia's market was quantified.

**Figure 3:**
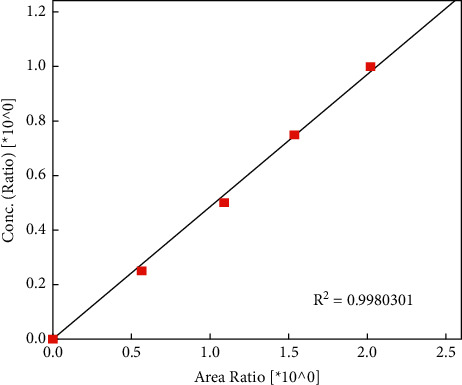
Group-calibration curve of hexane (0–68 mg kg^−1^).

According to AOCS [9], this official method is only suitable for the determination of quantities of hexane between 10 and 1500 mg kg^−1^. Hence, slight modification of the official AOCS method was made to improve the evaluation. Since the amount of residual hexane in edible oil often present in trace amount, which is outside the working range of the calibration curve (lowest standard is 67 mg kg^−1^), a new calibration curve with lower standard concentration (0–68 mg kg^−1^) was prepared as given in Table 2, in order to better match the low concentration of residual hexane in sample. Coefficient of determination (*R*^2^) value of more than 0.998 for the new calibration curve was obtained (Figure 3). Comparison of results between the official AOCS method with higher standard calibration concentration and the instrumental automated method with lower standard calibration concentrations was tabulated and is given in Table 3. Of all the four tested edible oils, only sunflower oil was found to contain the residual hexane that exceeded the maximum residue limit (MRL). Besides, the residual hexane detected in the large calibration range (0–938 mg kg^−1^) is significantly different as compared to the results from the small calibration range (0–68 mg kg^−1^). These results suggest that a large difference of calibration range might induce misinterpretation of analysis results while extrapolating from the calibration curve.

**Figure 4:**
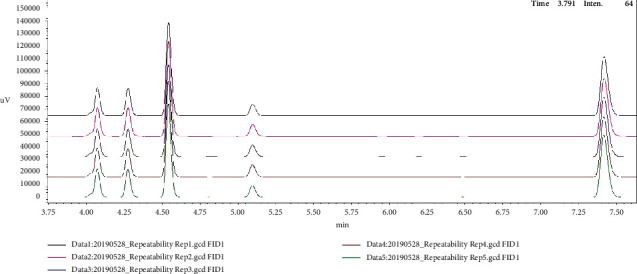
Chromatogram of hexane (34 mg kg^−1^) and internal standard (68 mg kg^−1^) showing the repeatability (*n* = 5).

**Figure 5:**
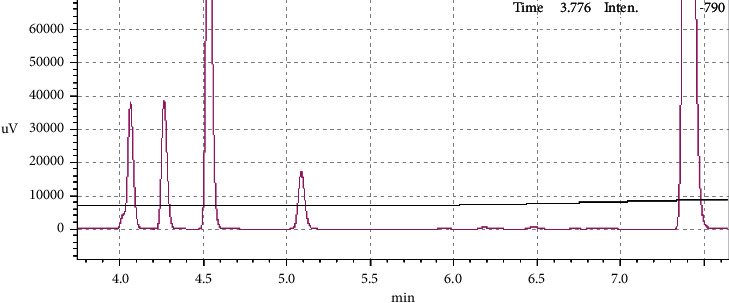
Chromatogram of standard showing low carryover check.

For method validation, performance checks have been performed. Theoretical quantitation limit (LOQ) based on the signal-to-noise (S/N) value was calculated using the Shimadzu LabSolutions software (Table 4). The repeatability of the system was tested using five different vials containing 5 g of oil spiked with 134 mg kg^−1^ of hexane and internal standard (ISTD). The average result obtained was 137 mg kg^−1^ (*n* = 5) with a low level of relative standard deviation (RSD) of 1.69% and 102% recovery (Figure 4). A measurement using blank was also performed to check for carryover. The result showed an extremely low carryover indicating the good system performance of Shimadzu HS-20 as well as the reliability of this method (Figure 5).

## 4. Conclusion

The sunflower oil was found to contain the residual hexane that exceeded the maximum residue limit (1 mg kg^−1^) among the four tested edible oils in Malaysia. In this analysis, optimization of the incubation time has drastically reduced half of the analysis time following the official AOCS Ca 3b-87 method. Besides, modification of the large calibration range to a lower calibration range (0–68 mg kg^−1^) in the calibration curve showed a significant difference compared to the results obtained following the official AOCS Ca 3b-87 method (0–938 mg kg^−1^). The static autoheadspace analysis method could be a recommended tool for the quantitative analysis method of residual hexane in vegetable liquid oil samples. With various automated features, this system has the capability to produce highly reliable quantification analysis. In conclusion, these results provide a fundamental reference for the improvement of the official AOCS Ca 3b-87 method in the future.

## Figures and Tables

**Table 1:** Amount of technical hexane added in five gram of oil.

Concentration	Vial number					
	1	2	3	4	5	6
*μ*l/5 g	0	0.5	1	2	4	7
mg kg^−1^	0	67	134	268	536	938

**Table 2:** Amount of technical hexane added in 20 g of oil.

Concentration	Vial number				
	1	2	3	4	5
*μ*l/5 g	0	0.5	1	1.5	2
mg kg^−1^	0	17	34	51	68

**Table 3:** Comparison of results of residual hexane in different edible oils.

Edible oils	Residual hexane concentration (mg kg^−1^)	
	AOCS official method with higher standard calibration concentration (0–938 mg kg^−1^)	Modified AOCS official method with lower standard calibration concentration (0–68 mg kg^−1^)
Sunflower oil	3.074	2.688^a^
Corn oil	0.414	0.358^a^
Sesame oil	0.711	0.602^a^
Palm oil	0.144	0.126^a^

^a^Significant difference at *p* < 0.05.

**Table 4:** Theoretical quantitation limit (LOQ) and signal-to-noise (S/N) based on 17 mg kg^−1^ standard.

Compound	S/N	LOQ (mg kg^−1^)
2-Methylpentane	218.20	0.85
3-Methylpentane	221.45	0.84
n-Hexane	746.37	0.25
Methylcyclopentane	97.37	1.93

## Data Availability

The data used to support the findings of this study are available at Shimadzu Malaysia Sdn Bhd.
